# Accelerated *In Vivo* Proliferation of Memory Phenotype CD4^+^ T-cells in Human HIV-1 Infection Irrespective of Viral Chemokine Co-receptor Tropism

**DOI:** 10.1371/journal.ppat.1003310

**Published:** 2013-04-18

**Authors:** Yan Zhang, Catherine de Lara, Andrew Worth, Andrea Hegedus, Karoliina Laamanen, Peter Beverley, Derek Macallan

**Affiliations:** 1 Infection and Immunity Research Centre, St George's, University of London, London, United Kingdom; 2 Nuffield Department of Medicine, University of Oxford, Oxford, United Kingdom; National Institute of Allergy and Infectious Diseases, National Institutes of Health, United States of America

## Abstract

CD4^+^ T-cell loss is the hallmark of HIV-1 infection. CD4 counts fall more rapidly in advanced disease when CCR5-tropic viral strains tend to be replaced by X4-tropic viruses. We hypothesized: (i) that the early dominance of CCR5-tropic viruses results from faster turnover rates of CCR5^+^ cells, and (ii) that X4-tropic strains exert greater pathogenicity by preferentially increasing turnover rates within the CXCR4^+^ compartment. To test these hypotheses we measured *in vivo* turnover rates of CD4^+^ T-cell subpopulations sorted by chemokine receptor expression, using *in vivo* deuterium-glucose labeling. Deuterium enrichment was modeled to derive *in vivo* proliferation (*p*) and disappearance (d*) rates which were related to viral tropism data. 13 healthy controls and 13 treatment-naive HIV-1-infected subjects (CD4 143–569 cells/ul) participated. CCR5-expression defined a CD4^+^ subpopulation of predominantly CD45R0^+^ memory cells with accelerated *in vivo* proliferation (*p* = 2.50 vs 1.60%/d, CCR5^+^ vs CCR5^−^; healthy controls; P<0.01). Conversely, CXCR4 expression defined CD4^+^ T-cells (predominantly CD45RA^+^ naive cells) with low turnover rates. The dominant effect of HIV infection was accelerated turnover of CCR5^+^CD45R0^+^CD4^+^ memory T-cells (*p* = 5.16 vs 2.50%/d, HIV vs controls; P<0.05), naïve cells being relatively unaffected. Similar patterns were observed whether the dominant circulating HIV-1 strain was R5-tropic (n = 9) or X4-tropic (n = 4). Although numbers were small, X4-tropic viruses did not appear to specifically drive turnover of CXCR4-expressing cells (*p* = 0.54 vs 0.72 vs 0.44%/d in control, R5-tropic, and X4-tropic groups respectively). Our data are most consistent with models in which CD4^+^ T-cell loss is primarily driven by non-specific immune activation.

## Author Summary

Loss of CD4^+^ T-cells is the cardinal feature of HIV/AIDS, resulting in pathological susceptibility to opportunistic infections. Mechanisms underlying CD4-depletion remain unclear, although the role of chronic immune activation is now well-recognized. Selectivity of the virus for its co-receptor target (either chemokine-receptor CCR5 or CXCR4) is also pivotal. We explored the relationship between these two factors by directly measuring *in vivo* proliferation rates of CD4^+^ T-cell subpopulations according to their expression of chemokine-receptors and the tropism of circulating virus in clinically-well people with HIV infection, and healthy human controls. We used stable isotope labeling with deuterium-labeled glucose to quantify proliferation and disappearance rate constants of CD4^+^ T-cells sorted by CCR5, CXCR4 and CD45R0/RA expression. We found that CCR5-expression defines a high turnover subpopulation which is therefore likely to be preferentially infected and produce more (CCR5-tropic) virus. CXCR4-tropic viruses induced a similar pattern of proliferation as R5-tropic strains, with no apparent selectivity for viral strains to induce proliferation in their targeted subpopulations. This study is significant in providing directly-measured *in vivo* human data supporting postulates generated in *ex vivo* human studies and SIV models suggesting that non-specific factors, such as immune activation, rather than cell-specific cytotoxicity, are dominant drivers for HIV pathogenesis.

## Introduction

The cardinal pathological feature of the acquired immunodeficiency syndrome (AIDS) is progressive CD4^+^ T cell depletion, but the immuno-pathological mechanisms linking chronic HIV infection with slow but progressive loss of CD4 cells, over periods measured in years, remain incompletely explained.[1] HIV preferentially infects CD4^+^ T cells, resulting in death of the host cell, but direct viral cytopathicity fails to adequately explain the kinetics and extent of CD4 loss.[2], [3] Other factors must be important and we now recognize altered immune homeostasis, immune activation and infection of gut lymphoid tissue as critical factors.

Any change in lymphocyte numbers must be considered in the context of immune homeostasis, the self-regenerative capacity of lymphoid populations. Homeostasis can be defined and measured in terms of three fluxes for each lymphocyte subset: proliferation, death and phenotype transformation. In uninfected individuals, these fluxes are balanced, maintaining roughly constant T-cell numbers for decades, and together these fluxes can be expressed as a “turnover” rate. Even in chronic-phase HIV-infected individuals, T-cell populations remain roughly stable on a day-to-day basis. Although CD4 cells are lost, loss rates are orders of magnitude less than everyday turnover, such that typical depletion rates represent a mismatch between proliferation and death of only ∼1%; hence even in progressive HIV-1 infection, at least 99% of dying lymphocytes are replaced on a daily basis. Proliferation may be either homeostatic or activation-induced; the latter tends to occur in bursts and, for naïve cells, is usually associated with phenotype change to “memory” phenotype. Such cells would thus be lost from the naïve compartment. However, in a homeostatic system, their loss will be matched by production of new naïve cells, predominantly in adult humans by proliferation within the peripheral compartment, as T-cell homeostasis continues unimpeded long after thymic involution.[4], [5]

Accelerated T-cell turnover [6]–[8] appears pivotal in causing retroviral-induced failure of T-cell homeostasis; thus the absence of a proliferative response in sooty mangabey SIV infection is associated with non-pathogenicity.[9] But what drives such turnover? Early paradigms invoked a homeostatic response to direct virus-mediated cell death.[10] However, this model alone cannot explain the loss of virtually the entire CD4+ compartment in advanced disease, not least because typically only about 1 in 1,000 CD4 cells are infected in untreated patients.[11], [12] Nor does it explain the correlation of CD8 and CD4 T-cell proliferation seen, for example, in bromodeoxyuridine (BrdU)-labeling studies.[13] Immune activation, by contrast, could affect the whole pool, and indeed correlates with disease progression,[14] Although HIV-specific cells are preferentially infected by HIV,[15] CD4^+^ activation is not confined to HIV-specific cells. Activation of a broad repertoire of cells may result from gastrointestinal microbial translocation [1] and/or expansion of the gastrointestinal virome [16] as a consequence of depletion of gastrointestinal lymphoid tissue, an early target of HIV infection,

However, immune activation *per se*, does not inevitably lead to CD4 depletion, as illustrated by other chronic inflammatory states. Failure of homeostatic replacement must also occur and may be related to cell-specific targeting by HIV infection. Viral entry into CD4+ T-cells requires specific binding to chemokine receptors alongside CD4, either CCR5 or CXCR4 for HIV-1. R5-tropic strains of virus predominate in early infection. It has been suggested that this relates to the abundance of CCR5-expressing macrophages at sites of primary infection, but an alternative explanation suggests that host cell division rates may determine the dominant viral type.[17], [18] According to this model, CCR5-expressing cells, predominantly memory/activated T-cells have higher rates of turnover,[19], [20] and thus produce more of the virus which preferentially infects them, in this case R5-tropic virus. Conversely, CXCR4-expressing cells are mostly naive cells, which are slowly-dividing,[19], [21] and so produce less (X4-tropic) virus.

Why then is the emergence of X4-tropic strains associated with accelerated disease progression and more dramatic loss of CD4 cells?[22], [23] We hypothesized that X4-tropic viruses may preferentially induce more immune activation and higher turnover in the (CXCR4^+^) cell populations they infect. Since these include predominantly naive cells, which are usually only replaced very slowly,[19], [21] the switch to X4-tropism may exceed compensatory immune homeostasis. If this is the case, *in vivo* CD4 kinetics will vary according to viral tropism: CCR5^+^ cells will be more affected by R5-tropic viruses whilst CXCR4^+^ cells will be more affected by X4-tropic viruses. The converse hypothesis, that non-specific effects, such as immune activation, which drive CD4 cell depletion are more marked with X4-tropic viruses,[24] would predict CD4 kinetics independent of viral tropism. We tested these hypotheses by measuring *in vivo* cell proliferation and disappearance rates using deuterated glucose incorporation.[19], [25] First, we investigated the relationship between chemokine receptor expression and turnover in defined CD4 memory and naïve cell populations in both healthy and HIV-infected subjects. Secondly, we investigated the effect of HIV infection on turnover rates of these chemokine receptor expression-defined populations of CD4^+^ T-cells. Thirdly we investigated whether the cell subtype specific effects of HIV infection were dependent upon the chemokine receptor tropism of the dominant circulating virus.

## Results

### Subject characteristics

*In vivo* CD4+ T-cell turnover measurements were made using deuterium glucose labeling in 13 healthy controls and 13 HIV-positive subjects (Table 1). Nine of the HIV-positive subjects had predominantly R5-tropic and four X-4 tropic virus. Groups were balanced for gender. HIV-positive subjects tended to be older than control subjects (mean 38 vs 25 years) and, as expected, had lower mean CD4 counts than control subjects (mean [range]: 435 [143–569] versus 779 [503–1182] cells/ul; P<0.001), but higher CD8 counts (1041 [340–2304] versus 441 [255–733] cells/ul; P<0.001; Table 1). One subject (RH07), who was initially assessed during a phase of rapid CD4 decline, was reevaluated 15 months after starting antiretroviral therapy when viral load was undetectable and CD4 count rising.

**Table 1:** Subject characteristics.

Identifier	Age	Gender	Viral Load	Log VL	CD4	CD4 slope^1^	CD8	Viral Tropism
	(years)		copies/ml		×10^6^ cells/L	×10^6^ cells/month	×10^6^ cells/L	
**Controls**								
C27RR	30	M	N/A	N/A	870	0	430	N/A
RC01	23	F	N/A	N/A	701	0	611	
RC02	23	M	N/A	N/A	698	0	478	N/A
RC03	35	F	N/A	N/A	680	0	392	N/A
RC04	20	F	N/A	N/A	1182	0	491	N/A
RC05	20	F	N/A	N/A	1159	0	733	N/A
RC06	31	M	N/A	N/A	643	0	373	N/A
RC07	22	M	N/A	N/A	993	0	449	N/A
RC08	24	M	N/A	N/A	831	0	280	N/A
RC09	26	M	N/A	N/A	560	0	557	N/A
RC10	25	F	N/A	N/A	574	0	416	N/A
RC11	22	M	N/A	N/A	503	0	255	N/A
RC12	26	M	N/A	N/A	731	0	266	N/A
**Mean**	**25.2**				**779**		**441**	
SD	4.5				219		139	
**HIV-positive**								
**R5-tropic**								
RH02	33	M	14,300	4.16	525	−7.8	1,817	**R5**
RH04	33	M	105,000	5.02	427	−12.9	851	**R5**
RH05	42	M	1,640	3.21	569	−1.4	858	**R5**
RH06	31	M	39,100	4.59	533	+1.4	1,179	**R5**
RH07	55	F	586,000	5.77	143	−16.1	340	**R5**
RH08	49	M	3,920	3.59	522	+0.4	599	**R5**
RH10	38	F	197	2.29	528	−2.0	592	**R5**
RH12	37	M	46,700	4.67	352	−4.1	1,155	**R5**
RH13	41	F	62,000	4.79	282	−5.6	727	**R5**
**X4-tropic**								
RH01	31	M	67,291	4.83	474	+1.9	2,304	**X4**
RH09	21	F	23,200	4.37	310	−5.9	1,046	**X4**
RH11	37	M	22,800	4.36	523	+3.3	1,189	**X4**
RH14	49	F	11,800	4.07	472	−4.2	877	**X4**
**All HIV-positive**								
**Mean**	**38.2**		**75,688**	**4.29**	**435**	**−4.1**	**1,041**	
SD	9.1		156,378	0.88	127	5.7	527	

N/A – not applicable;

1 assumed to be zero for controls.

### Cell turnover and HIV infection

When we investigated *in vivo* cell turnover in specific CD4^+^ memory and naïve T-cell subpopulations we made five key observations:

#### 1. CCR5^+^ expression defines a fast turnover subpopulation of CD4^+^ CD45R0^+^ memory cells

**Figure 1:**
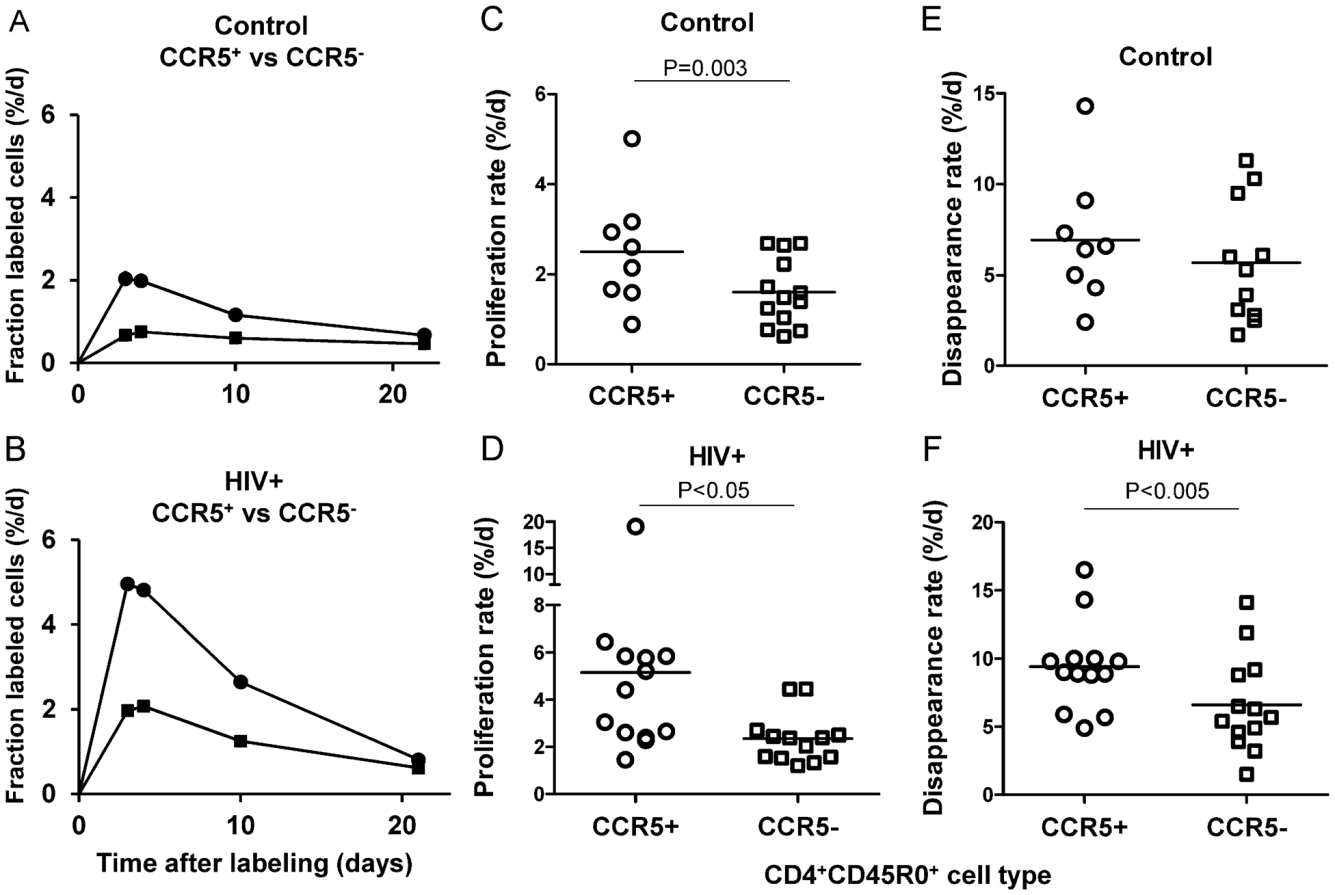
Relationship between CCR5 expression and proliferation, disappearance and phenotype in CD45R0^+^ CD4^+^ memory T-cells. (A,B) Deuterium enrichment plots expressed as fraction of labeled CD45R0^+^ CD4^+^ memory T-cells from a one-day labeling experiment. Cells are compared according to CCR5 expression: circles, CCR5^+^; squares, CCR5^−^. Representative data are shown from one typical control subject (A) and one HIV-positive subject (B). Modeled proliferation (C, D) and disappearance (E, F) rate constants for CD45R0^+^ memory CD4^+^ T-cell subsets defined by CCR5 expression in healthy (C,E) and HIV+ (D,F) subjects. Symbols denote cell type: circles, CCR5^+^; squares, CCR5^−^; horizontal bar, mean, P values by paired t-test.

Firstly, there was a clear relationship between memory cell turnover and CCR5 expression. In healthy subjects, CCR5 positive CD4^+^CD45R0^+^ T-cells had higher *in vivo* proliferation rates than CCR5-negative CD4^+^CD45R0^+^ cells (Figure 1A), when expressed either as peak enrichment values, which give a minimum estimate for turnover rate (Table S1, CCR5^+^, 2.02±0.67 vs CCR5^−^ 1.11±0.40%; P<0.001; mean ± 1SD and hereafter), or as modeled proliferation rates (*p*, Table 2 and Figure 1C, CCR5^+^, 2.50±1.26 vs CCR5^−^ 1.60±0.75%/d; P = 0.003). The same observation was made in HIV+ subjects; CCR5^+^ memory cells proliferated about twice as fast as CCR5^−^ memory cells (peak enrichment: Figure 1B, Table S1, CCR5^+^, 3.44±1.89 vs CCR5^−^ 1.87±0.78%, P = 0.016; proliferation rates: Table 2, Figure 1D, CCR5^+^, 5.16±4.51 vs CCR5^−^ 2.35±1.05%/d, P = 0.046). Disappearance rates (d*, Table S2, Figure 1E) consistently exceeded proliferation rates; this is expected in short-term labeling studies where disappearance rates only reflect the rate of loss of the small sub-fraction of recently-divided cells which inherently have high early post-labeling death-rates, whatever their phenotype or origin [19]. Despite this constraint and large inter-individual variation, we did observe an effect of CCR5-expression on the disappearance rates of labeled cells in HIV+ (but not control) subjects, CCR5^+^ cells disappearing from the circulation more rapidly than CCR5^−^ cells (Table S2; Figure 1F; P = 0.003).

**Table 2:** Modeled proliferation rates for CD4+ T-cell subpopulations.

	Cell phenotype					
	CD4+ CD45R0+ CCR5+	CD4+ CD45R0+ CCR5− CXCR4−	CD4+ CD45R0+ CCR5− (all)	CD4+ CD45R0+ CCR5− CXCR4+	CD4+ CD45R0− CXCR4−	CD4+ CD45R0− CXCR4+
**Controls**						
C27RR	n/a		1.40		0.95	0.20
RC01	n/a		1.48		0.76	
RC02	n/a		2.64		2.22	0.35
RC03	n/a		2.68		1.28	0.83
RC04	2.93		1.72		0.56	0.29
RC05	n/a		1.04		0.28	0.28
RC06	0.89		0.77		0.51	0.22
RC07	5.01		2.68		1.86	0.89
RC08	3.16		2.22		0.42	1.25
RC09	1.67		1.24		0.56	0.24
RC10	2.60		1.59		0.63	0.69
RC11	1.59		0.62		0.44	0.91
RC12	2.14		0.74		0.49	0.31
**mean**	**2.50** **		**1.60**		**0.84**	**0.54**
SD	1.26		0.75		0.59	0.36
n	8		13		13	12
**HIV-positive**						
**R5-tropic**						
RH02	4.42	2.16	2.03	1.94		0.51
RH04	5.77	1.61	1.21	1.61		1.10
RH05	5.22	2.66	2.50	0.67		0.53
RH06	5.85	1.90	1.52	1.97		0.81
RH07	19.07	2.07	2.39	2.64		1.02
RH08	2.66	1.60	1.59	0.76		0.36
RH10	2.28	2.51	2.45	1.98		0.72
RH12	1.46	1.71	1.57	0.92		0.82
RH13	3.05	4.73	4.45	1.24		0.64
**R5-mean**	**5.53**	**2.33**	**2.19**	**1.52**		**0.72**
R5-SD	5.32	0.97	0.97	0.67		0.24
*R5 HIV dynamic factor*	*2.21*		*1.37*			*1.34*
**X4-tropic**						
RH01	2.40	n/a	1.33	n/a	1.16	0.49
RH09	6.46	4.58	4.46	1.54		0.58
RH11	5.85	2.42	2.38	2.17		0.61
RH14	2.61	2.88	2.71	0.93		0.07
**X4-mean**	**4.33**	**3.29**	**2.72**	**1.55**		**0.44**
X4-SD	2.13	1.13	1.30	0.62		0.25
*X4 HIV dynamic factor*	*1.73*		*1.70*			*0.81*
**All HIV-positive**						
**mean**	**5.16** † *	**2.57** §	**2.35** §	**1.53** ¶		**0.63**
SD	4.51	1.24	1.05	0.74		0.27
*All HIV dynamic factor*	*2.07*		*1.47*			*1.18*

Values represent proportion of labeled cells newly generated per day, normalized for average glucose enrichment. CCR5^−^ cells were sorted by CXCR4 expression in all HIV subjects (except RH01) but not in controls. n/a, data not available. HIV dynamic factor represents the mean proportional effect of HIV infection on turnover rates compared to controls. In order to avoid multiple comparisons, values for proliferation rate were only compared within corresponding cell-types between subject groups (control versus “All HIV-positive” subjects, not with separate HIV-positive subgroups),

† P<0.05 versus control subjects for CCR5^+^ and.

§ P<0.05 for CCR5^−^ cells, unpaired t-test; within cell-type between HIV groups (no significant differences); and between corresponding cell-types (chemokine receptor positive versus negative) within subject groups,

* P<0.05,

** P<0.01 CCR5^+^ versus CCR5^−^ cells,

¶ P<0.05 CXCR4^+^ versus CXCR4^−^ CCR5^−^ cells, paired t-tests.

Since CCR5 expression appeared to define a memory T-cell subset with accelerated turnover in all subjects, we sought to further characterize such cells phenotypically. We found that CCR5-expression was associated with a more activated and differentiated phenotype, compared to CCR5-negative memory cells. Similar observations have been made in SIV-infected macaques.[26] When compared to CCR5^−^ cells, CCR5^+^ memory cells also had lower levels of CCR7, CD27 (in both control and HIV^+^ subjects), and CXCR4-expression, although in two HIV-infected subjects CXCR4 levels were increased (Figure S1). CCR5^+^ and CCR5^−^ cells exhibited similar levels of expression of CD25, CD28, and CD95. Most CCR5^+^ cells thus appear to have an effector-memory phenotype. Interestingly, in HIV subjects CD45R0^+^CXCR4^−^ cells had a significantly more differentiated phenotype, having lost more CD27 and CCR7 (Figure S1).

#### 2. HIV infection dramatically accelerates the turnover of CCR5^+^ memory (CD4^+^CD45R0^+^) CD4^+^ T cells

**Figure 2:**
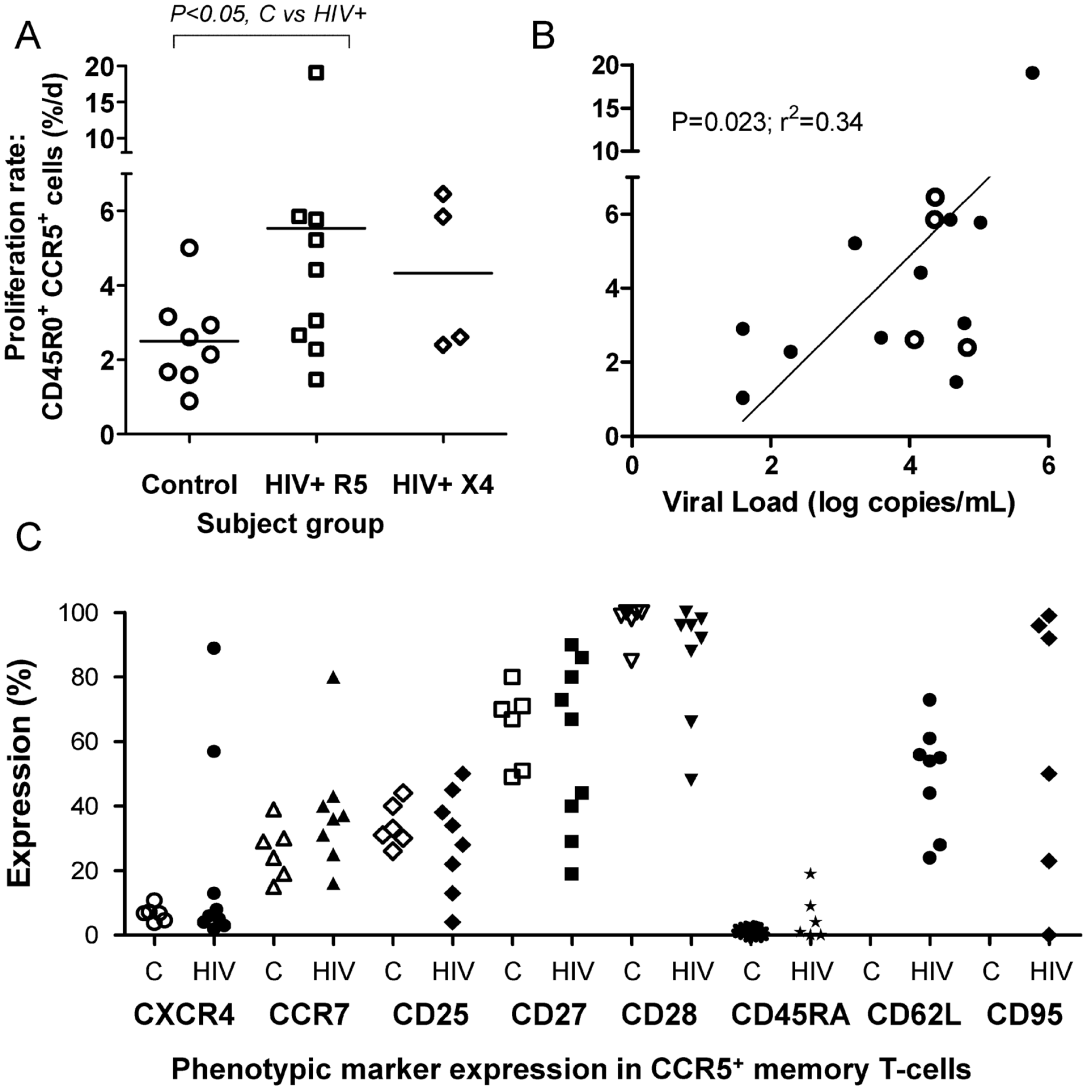
HIV infection accelerates turnover rates of CCR5-positive CD4^+^CD45R0^+^ memory T-cells. (A) Increased proliferation rates in HIV infected subjects (HIV+ R5, HIV-positive subjects with R5-tropic virus; HIV+ X4, HIV-positive subjects with X4-tropic virus) compared to controls (P<0.05 by Mann Whitney test as data skewed). (B) Correlation of CCR5^+^ T-cell turnover with viral load in viremic HIV+ subjects (open circles are subjects with X4-tropic virus; P = 0.044, r^2^ = 0.28 using log-transformed data). (C) Phenotype profile of CCR5^+^ memory T-cells in healthy controls (C, open symbols) and HIV+ subjects (H, filled symbols). CD62L and CD95 data were not available for control subjects. P = NS for all comparisons between control and HIV+ by Mann Whitney test.

The most marked effect of HIV on the kinetics of CD4^+^ T-cells was found in the CCR5^+^ subset where HIV infection more than doubled modeled proliferation rates (5.16±4.51 versus 2.50±1.26%/d, HIV+ vs controls, P = 0.046, ratio (HIV dynamic factor) 2.07,Table 2). This was also apparent as an increase in mean peak enrichment (Table S1; P<0.05). Although numbers of subjects with X4-tropic virus were small, the effect appeared similar for both viral tropism cohorts (Figure 2A). There was a trend towards higher CCR5^+^ memory cell (CD4^+^CD45R0^+^) turnover in subjects with higher viral loads (Figure 2B, P = 0.023).

Since the kinetics of CCR5^+^ cells differed between HIV^+^ and control subjects, we investigated whether this might be associated with a difference in the phenotype of such cells. This did not appear to be the case for several key phenotypic markers (Figure 2C). CD45R0^+^CCR5^+^ cells in HIV-infected subjects tended to be CD28^+^ (86%), CD27^+^ (59%), and CD95^+^ (60%), whilst exhibiting intermediate staining for CCR7 (39%), CD25 (29%), and CD62L (49%), and low levels of staining for CD45RA (5%) and CXCR4, except in two subjects (mean 31%). Similar patterns of co-staining for these markers, albeit with less variability, was observed on CCR5^+^ cells in control subjects (except CD62L and CD95 which were not analyzed in control subjects).

#### 3. HIV infection accelerates turnover of CCR5-negative CD4^+^CD45R0^+^ memory cells, predominantly in the CXCR4-negative subpopulation

**Figure 3:**
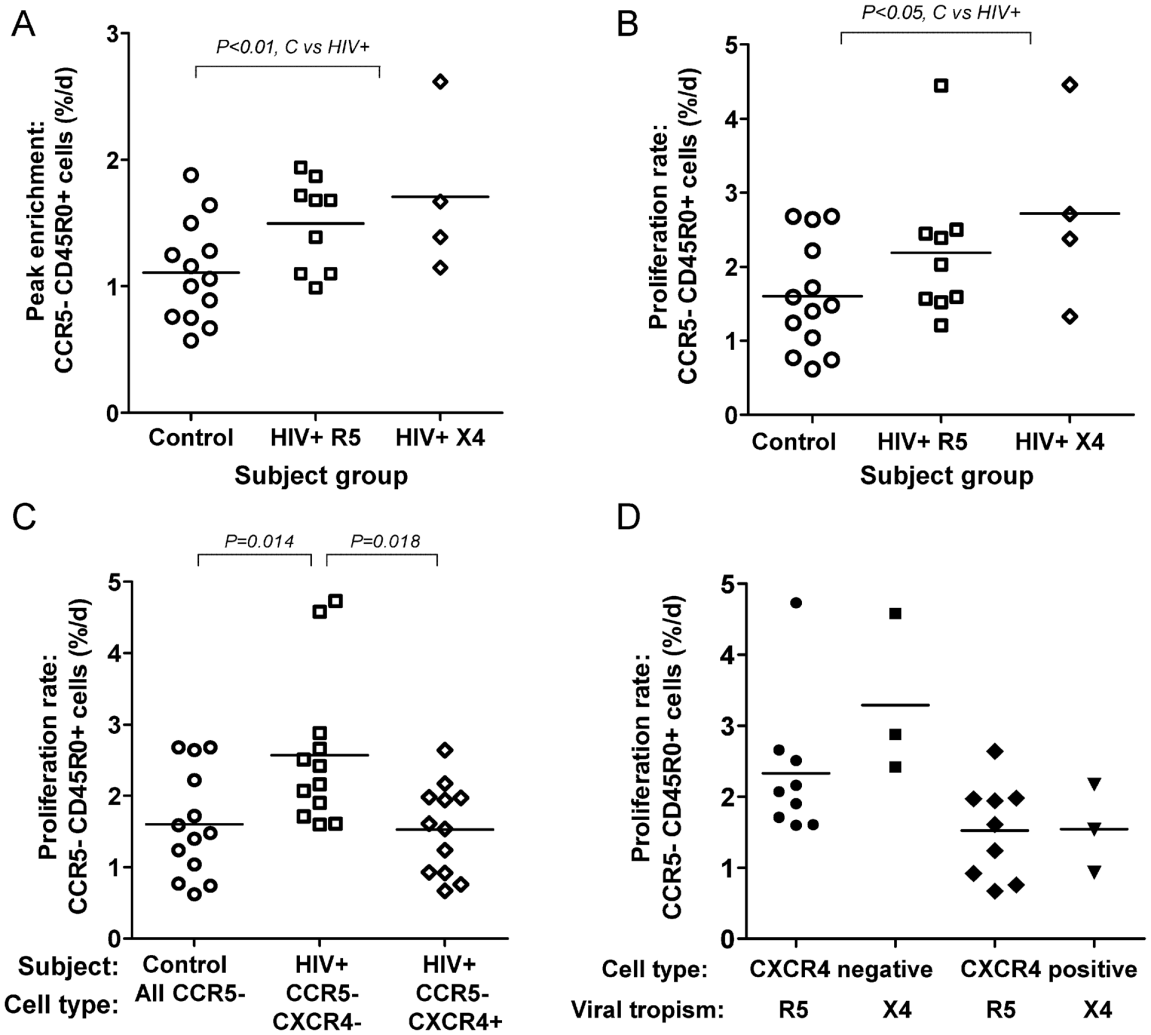
HIV infection increases turnover rates of CCR5-negative CD4^+^CD45R0^+^ memory T-cells. HIV infection and turnover rates of CCR5^−^ memory T-cells, expressed either as peak enrichment (A) or modeled proliferation rate (B) for all CD4^+^CD45R0^+^ CCR5^−^ cells, according to subject group: HIV+ R5, HIV-positive subjects with R5-tropic virus; HIV+ X4, HIV-positive subjects with X4-tropic virus. When CCR5^−^ cells in HIV+ subjects were subdivided by CXCR4 expression (C), the effect of HIV infection was found to be confined to the CXCR4^−^ compartment; x-axis separated by subject group and cell type; control samples were not subdivided by CXCR4 expression. (D) Relationship between proliferation rates, CXCR4 expression and viral tropism in CD4^+^CD45R0^+^ CCR5^−^ cells in HIV+ subjects.

When we investigated CCR5^−^ memory cells, we found that HIV also accelerated the turnover of such cells by a factor of 1.47 overall (Table 2), although, in absolute terms, they still proliferated less quickly than corresponding CCR5^+^ cells. Mean peak enrichment was increased by about 70% (1.87 vs 1.11%; P = 0.004; Figure 3A) and modeled proliferation rate by ∼50% (Figure 3B; P = 0.047). Disappearance rates were similar for both control and HIV-positive populations (Table S2).

We next tested whether the effect of HIV infection on CCR5^−^ cells was related to the interaction between CXCR4-receptor expression and viral tropism by sorting cells for CXCR4-receptor co-expression prior to deuterium analysis. We found high rates of proliferation in CXCR4-negative CCR5^−^ cells (Figure 3C, P = 0.018), whilst cells expressing CXCR4 had low rates of proliferation, similar to those in control CCR5^−^ cells (Figure 3C). Although this subgroup analysis was only performed in three subjects with an X4-tropic virus analyzed, there did not appear to be any preferential stimulation of proliferation in CXCR4^+^ memory cells in those with X4-tropic virus; conversely, proliferation appeared to be accelerated in CXCR4^−^ cells just as much in subjects with X4-tropic virus as in those with R5-tropic strains (Figure 3D), suggesting that there is no selective effect of X4-tropic viruses on CXCR4^+^ cell turnover within the memory compartment. When we investigated disappearance rates of CCR5^−^ cells according to CXCR4 expression, the CXCR4^−^ cells appeared to have a long survival in the circulation (Table S2).

#### 4. Naïve (CD45R0^−^) CD4^+^ T-cell turnover is slow, irrespective of CXCR4 expression status

**Figure 4:**
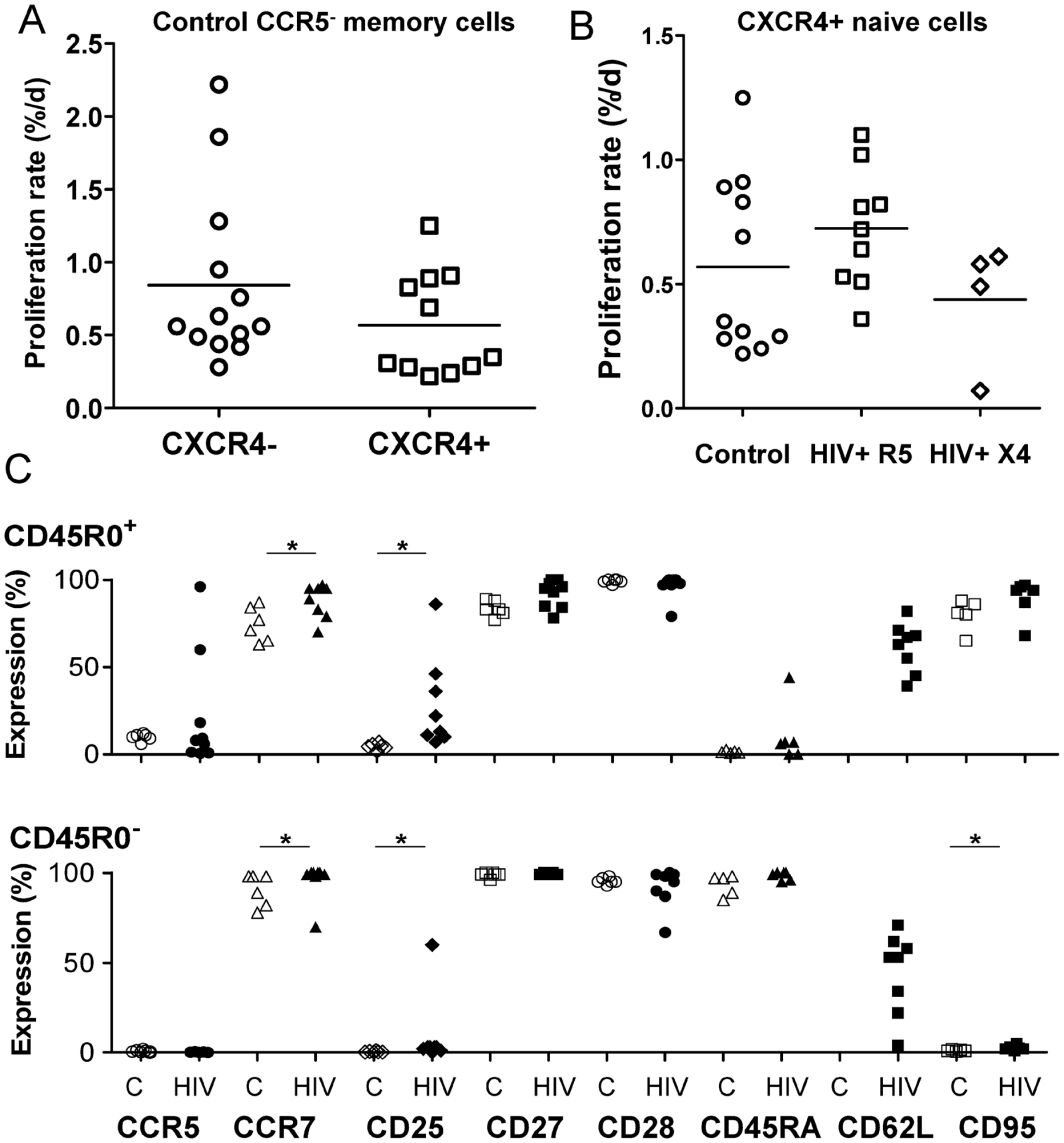
CXCR4 expression and turnover in CCR5^−^ memory (CD45R0^+^) T-cells, CXCR4^+^ naïve (CD45R0^−^) T-cells and phenotypic correlates. (A) Proliferation rates in CXCR4^−^ (circles) and CXCR4^+^ (squares) CCR5^−^ CD45R0^+^ T cells in healthy controls; horizontal bars represent means, P = NS by t-test. (B) Modeled proliferation rates in CXCR4^+^ naive (CD45R0^−^) CD4+T cells from control subjects (circles), HIV-infected subjects with an R5-tropic virus (squares), and HIV-infected subjects with an X4-tropic virus (diamonds); horizontal bars represent means, P = NS by t-test. (C) Phenotype of CXCR4^+^ cells according to CD45 expression, either CD45R0^+^ (upper panel) or CD45R0^−^ (lower panel). Values are shown from healthy control subjects (C, open symbols) and HIV+ subjects (H, closed symbols); * indicates significant difference between control and HIV data (P<0.05 by Mann Whitney test). CD62L data was not available for control subjects.

CXCR4 tends to be expressed on naïve T-cells rather than memory cells. Since it is known that naïve T-cells have much slower rates of *in vivo* turnover than memory cells,[19], [21] we hypothesized that CXCR4-expressing cells might represent a subset with slower turnover prior to HIV infection. We did not find evidence for such an effect in this small study (Figure 4A; P = NS); indeed turnover rates were low in all CD45R0^−^ cells in control subjects. This comparison was not possible in HIV^+^ subjects as CD45R0^−^ CXCR4^-^ were only collected in the HIV-sorting protocol in one subject.

#### 5. Effects of HIV infection on naïve cell turnover are minor, appear independent of viral tropism, and are associated with disease progression

When modeled naïve cell kinetics were compared between control and HIV+ groups, we found similar turnover rates for “naïve” CD45R0^−^CXCR4^+^CD4^+^ T-cells (Figure 4B; Table 2 and Table S1). Overall the effect of HIV infection on T-cell dynamics (a factor of 1.18±0.51) was not significant. Separating HIV-infected subjects according to viral tropism demonstrated that the presence of X4-tropic virus was not associated with any differential increase in proliferation of CXCR4^+^ cells. Indeed, although numbers were small, it appeared that the converse was true; CD45R0^−^CXCR4^+^ cells in subjects with X4-tropic viral strains tended to have lower, not higher, rates of proliferation (Table 2 and Table S1; Figure 4B) and disappearance (7.4 vs 11.8%/day, Table S2). Our findings thus appear contrary to the predictions of a direct viral pathogenicity model; X4 tropic viruses do not appear to preferentially drive turnover of CXCR4+ CD4+ T-cells. However, when we investigated the relationship between disease progression, using either viral load or CD4 slope as an index, we found that subjects with higher viral loads and more rapid CD4 loss had higher rates of proliferation within the naïve compartment, suggesting that with disease progression turnover within the naïve compartment is indeed accelerated.

In order to further define the effects of HIV on the CXCR4^+^ compartment, we performed extended phenotyping (Figure 4C). In controls, for CD45R0^+^ cells, CXCR4^+^ expression was associated with lower expression of CCR5 (11 vs 29%, medians, P = 0.036 by Wilcoxon test), and CD25 (4.6 vs 7.5%; P = 0.031). For CD45R0^−^ CXCR4^+^ cells, CCR5, CD25, and CD95 expression were all very low; low levels of CD95 would be consistent with greater longevity. CD28 expression levels tended to be high and unrelated to CXCR4 expression. HIV infection resulted in modest up-regulation of CD25 on all cell types, and of CD95 on CD45R0^−^ cells, whether CXCR4^+^ or negative (Figure 4C). A differential effect was seen in CD45R0^+^ cells with both CCR7 and CD27, where HIV increased expression of both markers in CXCR4^+^ cells but reduced it in CXCR4^−^ cells.

Thus it appears that CXCR4^+^ cells share many phenotypic characteristics regardless of their CD45R0/RA status (CD95 being an exception). Both subpopulations show a similar response to HIV infection, primarily increased activation as reflected by CD25 expression. Strikingly, however, HIV infection did not appear to drive differentiation of CXCR4^+^ cells (or at least not without transition to CXCR4-negativity) as CD27, CD28 and CCR7 expression remained high. Thus both phenotypic and kinetic data support the proposition that a small but significant number of cells are entering an activated state and cycling without leaving the CXCR4^+^ compartment, but only as disease progression occurs.

### Predictors of CD4 T-cell loss

**Figure 5:**
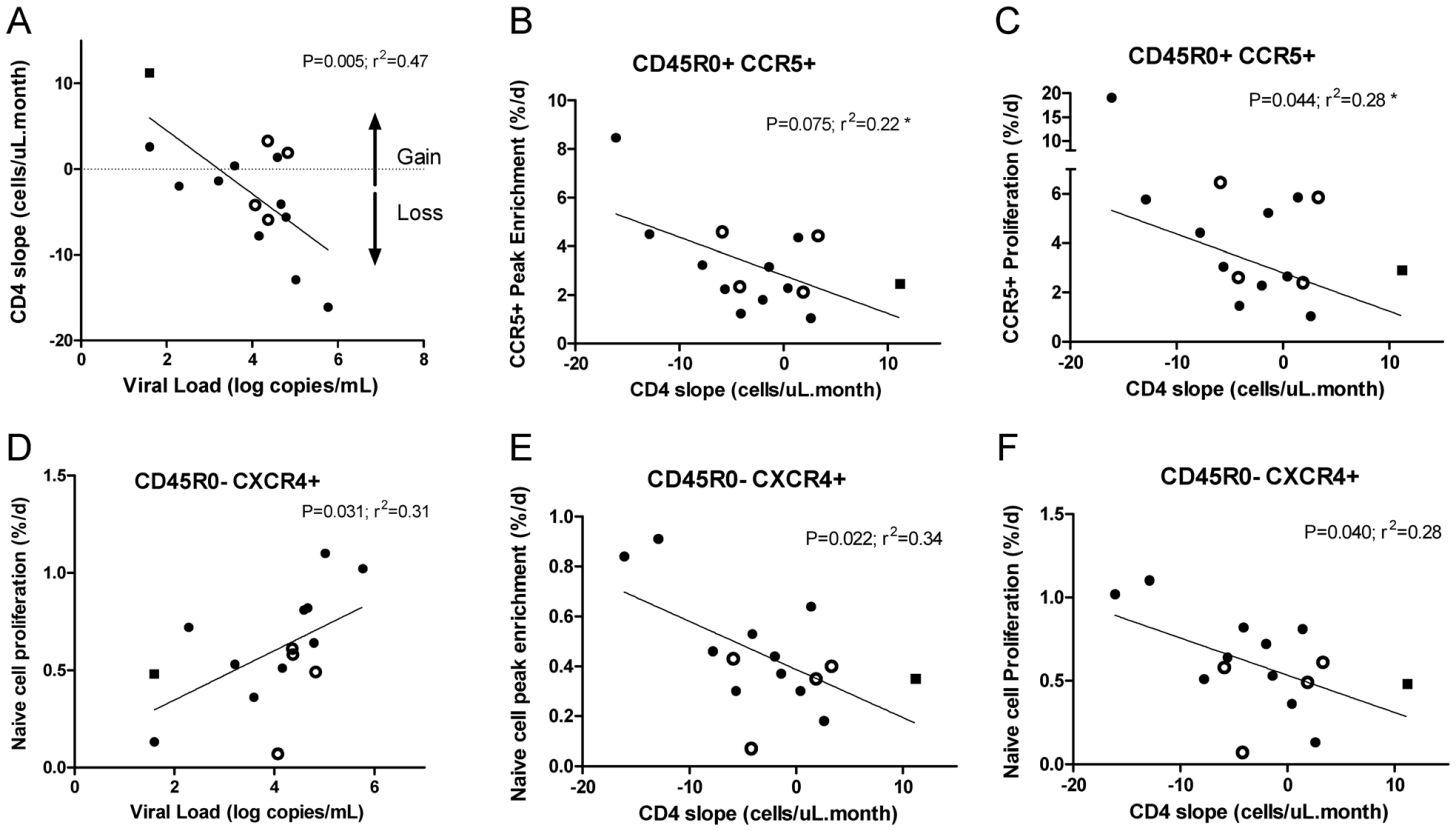
Correlates of CD4 cell loss in HIV-infected subjects. Relationship of CD4 slope with viral load in all HIV-infected subjects (A); each symbol represents a single subject; closed circles, R5-tropic subjects; open symbols, X4-tropic subjects; closed square, treated subject (RH07R); undetectable viral load was attributed the value of 40 copies/mL (the detection threshold), or 1.6 as log_10_. CD4 cell loss was expressed as a rate of decline in the months preceding *in vivo* kinetic studies. Relationship of viral load and CD4 slope with kinetic parameters in memory (CD45R0^+^CCR5^+^) (B, C) and naïve (CD45R0^−^CXCR4^+^) (D, E, F) CD4^+^ T-cells; symbols as frame (A); analysis by Pearson linear regression, * log transformed for skewed CCR5+ data.

In order to further elucidate the pathophysiology of T-cell loss in HIV infection we related kinetic parameters to the rate of change of CD4 count in the period leading up to the labelling day. As expected, there was a strong negative correlation between viral load and rate of change of CD4 count (Figure 5A; r^2^ = 0.47; P = 0.005). These two parameters can thus be considered covariate expressions of disease progression. Thus, the trend towards faster CCR5^+^ cell turnover with higher viral loads (Figure 2B) corresponded to similar trend with more rapid CD4 decline (Figure 5B & C). Correlation of markers of disease progression with turnover of CCR5^−^ memory cells was not significant in this small study, whereas there did appear to be a significant relationship with naïve cell turnover (Figure 5D,E,F), although data were strongly influenced by rapidly progressing subjects such as RH07. This subject was studied twice, initially during a phase of rapid CD4 cell loss and subsequently, 15 months after starting antiretroviral therapy, with an undetectable viral load (<40 copies/ml) and rising CD4 count (from 188 to 558 cell/ul; Figure S2A). Changes in turnover rates pre-/post-treatment mirrored the between cohort differences described above. The most dramatic change was a reduction in the turnover rate of CCR5^+^ memory cells, by a factor of more than 6-fold (Figure S2B,S2D), whereas changes in the turnover rate of CCR5^−^CXCR4^−^ memory cells were less marked. For CXCR4^+^ cells in both naïve and memory compartments, a two-fold reduction in peak labelling was also noted (Figure S2C, S2D), even though this patient had a predominantly R5-tropic circulating strain of virus, suggesting that the proliferation-inducing effects of HIV infection in this subject were not due to cellular tropism directed, subset-specific effects of viral infection.

## Discussion

Previous studies in SIV models [9], [27] and *ex vivo* studies in humans [28], [29] have greatly informed our understanding of lentivirus immunopathology. Although only a relatively small descriptive study, this research extends our understanding by adding directly-measured human *in vivo* data exploring how CD4 T-cell immune homeostatic responses contribute to HIV pathogenesis.

Firstly, we found that CCR5 expression normally defines a subpopulation of memory cells which divide more frequently than CCR5^−^ memory T-cells; this was true for both healthy and HIV-infected subjects. This is consistent with the observation that CCR5 is predominantly expressed on CD45RA^−^ antigen-primed memory cells which are known to have higher turnover rates than their naïve counterparts.[19], [21] In the context of HIV infection, this will render CCR5^+^ cells more susceptible to HIV infection and also more likely to be the source of circulating virus in infected individuals, consistent with a proliferation-based model of early R5-predominance.[17] Concurrently, CCR5^+^ cells represent the subpopulation whose turnover rate is most affected by HIV infection. By contrast, within the larger CCR5^−^ memory compartment, increased proliferation was only seen in the CXCR4^−^ compartment, possibly because activation is associated with downregulation of CXCR4 expression. Thus the effects of HIV infection are subpopulation specific. Similar observations have emerged from analogous studies in CD8 cells using heavy water labeling; HIV accelerated the turnover of IL-7Rα^+^ central-memory cells but not T_EMRA_ cells, which retained a long half-life and accumulated despite progressive HIV disease.[30]

Expression of CCR5 appears to be critically related to how cells respond to HIV infection. Upregulation of CCR5 may be both cause and effect. CCR5 would be expected to be upregulated on activated cells, but our phenotype data showed that most CCR5^+^ cells in HIV infection are not highly activated (little Class II or CD95), although they are more differentiated (reduced CD27). Greater differentiation may be associated with shorter inter-mitotic times as shown in previous studies of CD4 T-cells showing a hierarchy of division rates: T_EM_>T_CM_>T_N_,[20] although in this study HIV infection was not associated with down-regulation of CCR7 in CD45R0^+^CCR5^+^ cells. Thus it appears likely that both R5 and X4-tropic HIV strains preferentially drive accelerated turnover in the CCR5^+^ compartment (possibly by driving more cells into a CCR5-expressing phenotype) and promote greater differentiation within that compartment.

Secondly, we found that, by contrast, naïve (CD45R0^−^CXCR4^+^) T-cell homeostatic proliferation remained relatively unaffected by the maelstrom of immune activation and CCR5^+^ cell proliferation. This accords with observations in HIV-infected subjects monitored by Ki67 expression and *ex vivo* BrdU incorporation.[29] Similarly, in patients with acute infectious mononucleosis, we previously observed a similar phenomenon: massive alterations in CD8^+^CD45R0^+^ memory T-cell kinetics were associated with only minor changes in CD45RA^+^ cell kinetics.[31] Slow turnover CXCR4^+^ naïve cells thus appear relatively protected from apoptosis. These measurements however would not include activation-induced proliferation of naïve cells if such dividing cells down-regulated CXCR4 and upregulated CCR5; their deuterium labeling would be apparent in CCR5, not CXCR4-sorted populations. However, even if this were the case, in a relatively steady-state situation, as prevails in chronic-phase HIV, most CXCR4+ cells undergoing phenotype transition would be replaced. Assuming such replacement is primarily by peripheral cell division in adult subjects,[21] a labeling signal would still be expected in the CXCR4^+^ fraction, although it might be too small to detect in our relatively small control and HIV+ cohorts. Interestingly, within the HIV cohort a trend towards accelerated turnover with markers of disease progression suggests that the onset of accelerated proliferation in the naïve cell compartment may be a critical tipping-point beyond which decompensation occurs, precipitating more rapid CD4 cell decline.[32] . Naïve cells may be difficult to replace, as they have long inter-mitotic times [21] and replacement depends upon peripheral T-cell division, particularly in older people with reduced thymic output [5] (consistent with the faster HIV progression seen in older patients [33]). Although previous studies of Ki67 expression have suggested that naïve cells may be brought into cycle as a homeostatic response to falling CD4 cell numbers in HIV infection,[18], [34] we did not observe any correlation of naïve cell turnover with absolute CD4 count in this study (possibly because we selected subjects with relatively high CD4 counts) but we did observe a trend with rate of CD4 decline and viral load, surrogate markers of immune activation.

Thirdly, we noted similar patterns of kinetic response to HIV infection, regardless of viral tropism. Both viruses approximately doubled turnover of CCR5^+^ memory cells (*p* increased by a factor of of 2.2 and 1.7 for R5 and X4 viruses respectively, Table 2). We found no evidence of tropism-specific accelerated turnover in the CXCR4^+^ compartment for either memory or naïve cells in subjects with dominant X4-tropic infections.Rather, the dominant effect of X4-tropic virus was to drive accelerated turnover of CCR5^+^ cells, as seen with R5-tropic strains. This pattern is similar to that seen in BrdU-labeling studies in SIV-infected rhesus macaques, where R5 and X4-tropic viral strains both accelerated memory cell turnover.[27] This does not rule out a specific effect of this virus type on CXCR4^+^ cells as numbers of X4-infected subjects were small, primarily because patients with X4-tropic virus not embarking on treatment are uncommon and difficult to identify and recruit. However, it should be noted that, although subject numbers were small, when both repeated measurements over time and replicates at each time-point are considered, our conclusions are based on 1,585 enrichment measurements. Also, these findings are based on direct *in vivo* measurements, which although difficult and complex to execute, give a genuine representation of human *in vivo* HIV pathology. To minimise staining artefacts we only sorted fresh (not frozen) cells, and used sodium azide to block co-receptor internalisation.[35] It would have been very helpful to have sorted further cell types, such as CXCR4^+^ and CXCR4^−^ CD45R0^+^CCR5^−^ subsets from control subjects and CD8 T-cells; the latter would have been particularly valuable since CD8 cells may respond quite differently to HIV.[28], [36] However, these additional sorts were not possible within the clinical and technical constraints of the project.

Why then does X4 switching appear more pathogenic? Recent studies of viral env C2V3 sequences have shown that, although there appears to be little viral compartmentalization among cell subsets, switching coreceptor usage to X4 results in dramatically increased infection in naïve cells.[37] If this exceeded homeostatic mechanisms, then the switch from a R5- to a X4-tropic virus may indeed represent a pressure that may lead to a slow erosion of these cells. Mechanistically, IL-7, a type-I cytokine, may have a critical role in such a switch as levels correlates with CD4 T-cell depletion.[28], [38] IL-7 also appears to increase CD4^+^ T-cell surface CXCR4 density, HIV-1 production and the risk of emergence of X4-tropic strains of virus.[39] In addition, non-specific activation by cytokines, superantigens, or antigen-specific recruitment from gut microbial translocation might also drive naïve cells into clonal expansion. If X4 tropism generated greater degrees of immune activation,[24] it might explain the positive correlations observed between CD4 disappearance rate and CXCR4^+^ naïve cell proliferation that we observed (Figure 5 E,F).

In conclusion, our directly-measured *in vivo* human kinetic data lend further support to an immune activation model of HIV immunopathogenesis. We hypothesise that the dominant and early effect of HIV infection is to drive the proliferation of CCR5^+^ CD4 T-cells, independent of viral tropism, although X4-tropic strains may generate higher levels of immune activation. Such CCR5^+^ memory cells are normally relatively short-lived and may therfore be relatively “easy” to replace. In progressive or late-stage disease, more naïve cells are driven into cycle, by immune activation resulting from virus of either tropism. Such naïve cells, with their low natural rates of turnover and in the presence of thymic atrophy characteristic of adult humans, are difficult to replace and cumulative CD4 depletion results. Further kinetic studies could be designed to address this proposal, focusing on patients with more advanced disease.

## Materials and Methods

### Subjects

#### Ethics statement

Subjects gave written informed consent to protocols approved by local ethics committees (Wandsworth Research Ethics Committee) and study procedures were performed in accordance with the principles of the declaration of Helsinki.

Asymptomatic HIV-1-positive subjects were recruited from viremic treatment-naïve patients attending an urban HIV clinic; one aviremic long-term non-progressor was also included in the regression analyses. Healthy controls were recruited by local advertisement. Exclusions were: age <18 or >65, pregnancy, any active medical condition or opportunistic infection. Baseline assessment included clinical review, quantitative CD4 count (Trucount, BD Biosciences), lymphocyte phenotyping and viral load assay. CD4 slope was calculated by defining the period of preceding documented clinical stability (up to 30 months) from clinical records, then linear regression of CD4 counts during the stable period; this yielded 4–8 time-points per subject over a median period of 16 months (range 7–30 months). The immune phenotype of HIV-positive subjects was analyzed according to expression of CD4, CD45RA, CD45R0, CXCR4, CCR5, CCR7, CD25, CD27, CD28, CD62L, and CD95 on a BD FACS Calibur using the following antibodies (sourced from Becton, Dickinson U.K. Limited, Oxford, UK, unless stated otherwise): CD4-Biotin (ABD Serotec, Oxford, UK)/Streptavidin PerCp, CD45RO APC (Caltag-Medsystems Ltd, Towcester, UK), CXCR4 (CD184)-PE, CCR5 (CD195)-FITC, CD45RA PE & FITC, Purified mouse anti-Human CCR7/Goat anti-mouse IgM PE & FITC (SouthernBiotech, Birmingham, AL), CD25-PE, CD25-FITC (ABD Serotec), CD27 PE & FITC, CD28 PE & FITC, CD62L PE & FITC, CD95 PE &FITC. Viral tropism of the dominant circulating viral strain was determined by sequencing the HIV-1 gp120 V3-loop region using the geno2pheno algorithm (http://www.geno2pheno.org), the concordance of which with gold-standard tests is good, especially with higher CD4 cell counts and non-exposure to antiretroviral therapy;[40] in one case where PCR failed, the HIV Trofile assay was used (Monogram Biosciences, San Francisco, CA).

### Labeling methodology

In order to measure the proliferation and disappearance rates of lymphocyte subpopulations, subjects received a labeling dose of 60 g of 6,6-^2^H_2_-glucose (Cambridge Isotopes, MA) intravenously over 24 hours as previously described.[19], [25] Samples taken during labeling showed an average plasma glucose enrichment of 26% (range: 16–35%) over 24 hours. Follow-up heparinized blood samples were taken on days 3,4,10 and 21 post-labeling to estimate DNA enrichment; sodium azide (final concentration 0.2%w/v) was added to prevent internalization of chemokine-receptors between venepuncture and flow-cytometry.[35] Peripheral blood mononuclear cells (PBMC) were prepared by density centrifugation and sorted according to expression of CD4, CD45R0, CXCR4, and CCR5 by flow-cytometry as shown in Figure S3 (Moflow, Cytomation, Fort Collins, CO). Sorted cells underwent DNA extraction, digestion and derivatization for analysis by gas chromatography mass spectrometry (GC/MS) by negative chemical ionization of the pentafluoro tri-acetate derivative of deoxyadenosine for deuterium enrichment as previously described.[41]

### Modeling, comparisons and statistics

Peak incorporation of deuterium into DNA of labeled cells was taken as a minimum estimate of proliferation; by dividing by the corrected precursor glucose enrichment [25] this may be expressed as the proportion of labeled cells for a one-day labeling period. Deuterium enrichment data were modeled using a single exponential accrual and loss function as described by Asquith et al [19], [42] in Sigmaplot (Systat Software Inc, San Jose, CA), where fractional deuterium enrichment, F is given by:
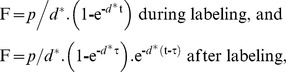
where t is the sample time and τ is the labeling time. This model yields two parameters: (i) the proliferation rate (*p*) represents the rate of replacement of the whole population of interest with newly synthesized cells and thus indicates population turnover, and (ii) the disappearance/death rate (d*) which contributes to the modeled *p* value but itself only describes the recently-divided sub-fraction of cells. Proliferation and disappearance rates were related to surface co-receptor expression and viral phenotype. Values are shown as mean ±1SD, except where ranges are shown [min-max].To avoid multiple comparisons, statistical comparisons were made according to an *a priori* plan (Table 2) using parametric tests (except where data failed D'Agostino-Pearson normality testing, when log-transformed data was used) in Prism (GraphPad Software Inc. La Jolla, CA).

## Supporting Information

Figure S1 **Phenotypic characteristics of CCR5^+^ and CCR5^−^ CD4^+^ T-cells.** Comparison of expression of CD27, CXCR4 and CCR7 on CCR5^+^ and CCR5^−^ CD4^+^ T-cells in control and HIV-positive subjects; not all control subjects in this group were fully immunophenotyped so further controls were included for comparisons of expression profiles. * indicates significant differences between CCR5^+^ and CCR5^−^ cells or between CXCR4^+^ and CXCR4^−^ cells; *P<0.05, **P<0.001 by paired t-test); † P<0.05 control vs corresponding HIV positive cell type.
(TIF)

Figure S2 **Effect of anti-retroviral treatment on turnover of CD4+ T cell subpopulations.** (A) Viral load (open squares) and CD4 count (closed circles) in subject RH07, who was infected with an R5-tropic virus, measured before (arrow “Pre-treatment”) and after (“Post-treatment”) commencing antiretroviral treatment (date shown by dashed line). (B & C) Corresponding patterns of turnover of CD4 T cells before (open symbols) and after treatment (solid symbols). Graphs show deuterium enrichment of DNA from sorted cell populations (expressed as fraction of new cells per day) for CD45R0^+^ memory CD4^+^ T-cells (B), subdivided into CCR5^+^ (diamonds) and CCR5^−^ (squares) subpopulations, and CXCR4 expressing cells (C, note different y-scale), subdivided into memory (CD45R0^+^, triangles) and naïve (CD45R0^−^, circles) subpopulations. (D) Tabulated changes in turnover rates of subpopulations.
(TIF)

Figure S3 **Sorting strategy.** Monoclonal antibody-labeled PBMC were sorted on a MoFlo, allowing simultaneous collection of four populations. (A) The lymphocyte gate was set using forward and side scatter parameters and cells were gated on CD4 (B) and then CD450 versus CXCR4 or CCR5 (C, D).
(TIF)

Table S1 **Peak enrichments (minimum proliferation rates) for CD4+ T-cell subpopulations.**
(DOC)

Table S2 **Modeled disappearance rates for labeled cells for CD4+ T-cell subpopulations.**
(DOC)
