# Supplementary figures and images for: Accelerated In Vivo Proliferation of Memory Phenotype CD4+ T-cells in Human HIV-1 Infection Irrespective of Viral Chemokine Co-receptor Tropism
Source: PLoS Pathog. 2013 Apr 18;9(4):e1003310. doi: 10.1371/journal.ppat.1003310 (PMC3630096; doi:10.1371/journal.ppat.1003310)

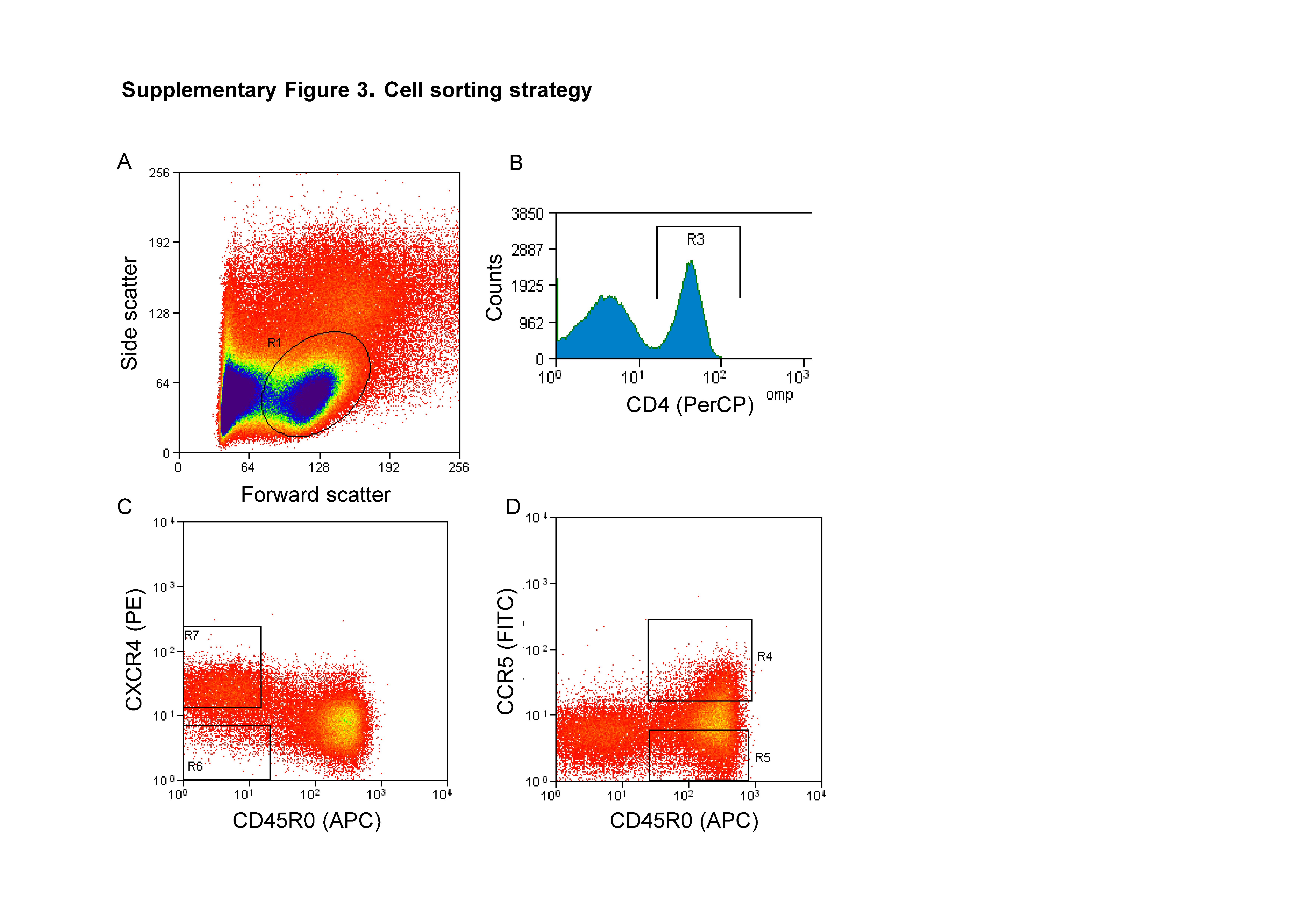

Supplement: Figure S3 — Sorting strategy. Monoclonal antibody-labeled PBMC were sorted on a MoFlo, allowing simultaneous collection of four populations. (A) The lymphocyte gate was set using forward and side scatter parameters and cells were gated on CD4 (B) and then CD450 versus CXCR4 or CCR5 (C, D). (TIF) [file ppat.1003310.s003.tif]
