# Supplementary material for: Accelerated In Vivo Proliferation of Memory Phenotype CD4+ T-cells in Human HIV-1 Infection Irrespective of Viral Chemokine Co-receptor Tropism
Source: PLoS Pathog. 2013 Apr 18;9(4):e1003310. doi: 10.1371/journal.ppat.1003310 (PMC3630096; doi:10.1371/journal.ppat.1003310)
Supplement: Table S1 — Peak enrichments (minimum proliferation rates) for CD4+ T-cell subpopulations. (DOC) [file ppat.1003310.s004.doc]

**Table S1. Peak enrichments (minimum proliferation rates) for CD4^+^ T-cell subpopulations**

| **Phenotype** | **CD4+ CD45R0+ CCR5+** | **CD4+ CD45R0+ CCR5- CXCR4-** | **CD4+ CD45R0+ CCR5-** | **CD4+ CD45R0+ CCR5- CXCR4+** | **CD4+ CD45R0- CXCR4-** | **CD4+ CD45R0- CXCR4+** |
| --- | --- | --- | --- | --- | --- | --- |
| **Controls** |  |  |  |  |  |  |
| C27RR | n/a |  | 1.64 |  | 0.65 | 0.10 |
| RC01 | n/a |  | 1.00 |  | 0.53 | n/a |
| RC02 | 2.17 |  | 1.16 |  | 1.46 | 0.19 |
| RC03 | 1.87 |  | 0.76 |  | 1.01 | 0.76 |
| RC04 | 2.46 |  | 1.50 |  | 0.34 | 0.32 |
| RC05 | n/a |  | 0.89 |  | 0.28 | 0.47 |
| RC06 | 0.84 |  | 0.75 |  | 0.47 | 0.30 |
| RC07 | 3.04 |  | 1.88 |  | 1.35 | 0.76 |
| RC08 | 2.73 |  | 1.28 |  | 0.40 | 0.95 |
| RC09 | 2.01 |  | 1.06 |  | 0.56 | 0.24 |
| RC10 | 2.08 |  | 1.25 |  | 0.63 | 0.47 |
| RC11 | 1.14 |  | 0.57 |  | 0.27 | 0.58 |
| RC12 | 1.81 |  | 0.67 |  | 0.41 | 0.22 |
| **mean** | **2.02**** |  | **1.11** |  | **0.64** | **0.45** |
| SD | **0.67** |  | 0.40 |  | 0.39 | 0.27 |
| n | **10** |  | 13 |  | 13 | 12 |
|  | |  |  |  |  |  |
| **HIV-positive** | |  |  |  |  |  |
| **R5-tropic** |  |  |  |  |  |  |
| RH02 | 3.23 | 1.80 | 1.68 | 1.33 |  | 0.46 |
| RH04 | 4.49 | 1.38 | 1.10 | 1.25 |  | 0.91 |
| RH05 | 3.15 | 2.15 | 1.87 | 0.49 |  | 0.37 |
| RH06 | 4.35 | 1.57 | 1.39 | 1.03 |  | 0.64 |
| RH07 | 8.46 | 1.66 | *1.69* | 1.71 |  | 0.84 |
| RH08 | 2.28 | 1.40 | *1.39* | 0.59 |  | 0.30 |
| RH10 | 1.80 | 2.05 | *1.97* | 1.39 |  | 0.44 |
| RH12 | 1.23 | 1.40 | *1.29* | 0.80 |  | 0.53 |
| RH13 | 2.24 | 3.19 | *2.99* | 0.69 |  | 0.30 |
| **mean** | **3.47** | **1.84** | **1.71** | **1.03** |  | **0.53** |
| SD | 2.17 | 0.58 | 0.56 | 0.42 |  | 0.22 |
| **X4-tropic** |  |  |  |  |  |  |
| RH01 | 2.11 | n/a | 1.15 | n/a | 0.76 | 0.35 |
| RH09 | 4.59 | 3.91 | *3.81* | 1.32 |  | 0.43 |
| RH11 | 4.42 | 1.85 | *1.68* | 0.93 |  | 0.40 |
| RH14 | 2.34 | 2.44 | *2.30* | 0.90 |  | 0.07 |
| **mean** | **3.37** | **2.73** | **2.23** | **1.05** |  | **0.31** |
| SD | 1.32 | 1.06 | 1.15 | 0.23 |  | 0.17 |
| **All HIV-positive** | |  |  |  |  |  |
| **mean** | **3.44*^†^** | **2.07** | **1.87^†^** | **1.04^¶^** |  | **0.46** |
| SD | 1.89 | 0.94 | 0.78 | 0.45 |  | 0.23 |
|  |  |  |  |  |  |  |

Values represent percentage of labeled cells normalized to a one day labeling period, equivalent to a minimum estimate of proliferation rate in %/day. CCR5^-^ cells were sorted according to CXCR4 expression in all HIV subjects (except RH01) but not in controls.

n/a, data not available. In order to avoid multiple comparisons, values for proliferation rate were only compared within corresponding cell-types between subject groups (control versus “All HIV-positive” subjects, not with separate HIV-positive subgroups), ^†^ P<0.05 versus control subjects for CCR5^+^ cells, unpaired t-test; within cell-type between HIV groups (no significant differences); and between corresponding cell-types (chemokine receptor positive versus negative) within subject groups,*P<0.05, **P<0.01 CCR5^+^ versus CCR5^-^ cells, ^¶^ P<0.05 CXCR4^+^ versus CXCR4^-^ for CCR5^-^ cells, paired t-tests.
