# Supplementary material for: Accelerated In Vivo Proliferation of Memory Phenotype CD4+ T-cells in Human HIV-1 Infection Irrespective of Viral Chemokine Co-receptor Tropism
Source: PLoS Pathog. 2013 Apr 18;9(4):e1003310. doi: 10.1371/journal.ppat.1003310 (PMC3630096; doi:10.1371/journal.ppat.1003310)
Supplement: Table S2 — Modeled disappearance rates for labeled cells for CD4+ T-cell subpopulations. (DOC) [file ppat.1003310.s005.doc]

**Table S2. Modeled disappearance rates for labeled cells for CD4+ T-cell subpopulations**

| **Phenotype** | **CD4+ CD45R0+ CCR5+** | **CD4+ CD45R0+ CCR5- CXCR4-** | **CD4+ CD45R0+ CCR5-** | **CD4+ CD45R0+ CCR5- CXCR4+** | **CD4+ CD45R0- CXCR4-** | **CD4+ CD45R0- CXCR4+** |
| --- | --- | --- | --- | --- | --- | --- |
| **Controls** |  |  |  |  |  |  |
| C27RR | n/a |  | 2.5 |  | 19.0 | 18.9 |
| RC01 | n/a |  | 11.3 |  | 10.6 | 3.9 |
| RC02 | n/a |  | n/a |  | 16.8 | 28.2 |
| RC03 | n/a |  | n/a |  | 9.1 | 5.4 |
| RC04 | 5.0 |  | 3.9 |  | 17.3 | 0.0 |
| RC05 | n/a |  | 6.1 |  |  | 0.0 |
| RC06 | 2.4 |  | 1.7 |  | 2.7 | 1.6 |
| RC07 | 14.3 |  | 10.3 |  | 11.4 | 8.0 |
| RC08 | 4.3 |  | 9.5 |  | 1.4 | 8.0 |
| RC09 | 6.4 |  | 6.0 |  | n/a | n/a |
| RC10 | 7.3 |  | 5.3 |  | 1.8 | 13.7 |
| RC11 | 9.1 |  | 2.8 |  | 20.5 | 18.0 |
| RC12 | 6.6 |  | 3.1 |  | 3.9 | 12.2 |
| **mean** | **6.9** |  | **5.7** |  | **10.4** | **9.0** |
| SD | 3.6 |  | 3.3 |  | 7.2 | 8.6 |
| n | 8 |  | 11 |  | 11 | 11 |
|  | |  |  |  |  |  |
| **HIV-positive** | |  |  |  |  |  |
| **R5-tropic** |  |  |  |  |  |  |
| RH02 | 9.8 | 4.6 | 5.4 | 11.1 |  | 3.3 |
| RH04 | 8.8 | 3.7 | 3.2 | 7.4 |  | 10.1 |
| RH05 | 14.3 | 6.7 | 8.8 | 8.3 |  | 10.3 |
| RH06 | 8.9 | 5.1 | 4.9 | 12.2 |  | 6.7 |
| RH07 | 16.5 | 8.4 | *14.1* | 18.6 |  | 8.7 |
| RH08 | 9.0 | 3.9 | *3.9* | 4.1 |  | 7.6 |
| RH10 | 10.0 | 6.0 | *6.5* | 10.4 |  | 19.2 |
| RH12 | 4.9 | 5.9 | *6.3* | 8.2 |  | 19.1 |
| RH13 | 8.9 | 11.5 | *11.9* | 16.7 |  | 20.8 |
| **mean** | **10.1** | **6.2** | **7.2** | **10.8** |  | **11.8** |
| SD | 3.4 | 2.5 | 3.7 | 4.6 |  | 6.3 |
| **X4-tropic** |  |  |  |  |  |  |
| RH01 | 5.7 |  | 1.5 |  | 18.8 | 9.3 |
| RH09 | 9.8 | 5.8 | *5.7* | 4.2 |  | 9.1 |
| RH11 | 10.0 | 8.1 | *9.2* | 13.9 |  | 11.2 |
| RH14 | 5.9 | 4.9 | *4.6* | 1.9 |  | 0.0 |
| **mean** | **7.8** | **6.3** | **5.2** | **6.7** |  | **7.4** |
| SD | 2.4 | 1.7 | 3.2 | 6.4 |  | 5.0 |
| **All HIV-positive** | |  |  |  |  |  |
| **mean** | **9.4*** | **6.2** | **6.6** | **9.7**^§^**^¶^** |  | **10.4** |
| SD | 3.2 | 2.7 | 3.5 | 5.6 |  | 6.1 |
|  |  |  |  |  |  |  |

Values are proportion (%) of labeled cells disappearing / dying per day. In order to avoid multiple comparisons, values for disappearance rate were only compared within corresponding cell-types between subject groups (control versus “All HIV-positive” subjects, not with separate HIV-positive subgroups), ^§^ P<0.05 versus control subjects for CCR5^-^ cells, unpaired t-test; within cell-type between HIV groups (no significant differences); and between corresponding cell-types (chemokine receptor positive versus negative) within subject groups,*P<0.01 CCR5^+^ versus CCR5^-^ cells, ^¶^ P<0.01 CXCR4^+^ versus CXCR4^-^ CCR5^-^ cells, paired t-tests.
